# Use of CRISPR-Cas9 To Target Homologous Recombination Limits Transformation-Induced Genomic Changes in Candida albicans

**DOI:** 10.1128/mSphere.00620-20

**Published:** 2020-09-02

**Authors:** Timea Marton, Corinne Maufrais, Christophe d’Enfert, Melanie Legrand

**Affiliations:** a Institut Pasteur, INRA, Unité Biologie et Pathogénicité Fongiques, Paris, France; b Université Paris Diderot, Sorbonne Paris Cité, Paris, France; c Hub de Bioinformatique et Biostatistique, Département de Biologie Computationnelle, USR 3756 IP CNRS, Institut Pasteur, Paris, France; University of Georgia

**Keywords:** CRISPR-Cas9, *Candida albicans*, genome rearrangements

## Abstract

Most of our knowledge relating to molecular mechanisms of human fungal pathogenesis in Candida albicans relies on reverse genetics approaches, requiring strain engineering. DNA-mediated transformation of C. albicans has been described as highly mutagenic, potentially accentuated by the organism’s genome plasticity, including the acquisition of genomic rearrangements, notably upon exposure to stress. The advent of CRISPR-Cas9 has vastly accelerated the process of genetically modifying strains, especially in diploid (such as C. albicans) and polyploid organisms. The effects of unleashing this nuclease within the genome of C. albicans are unknown, although several studies in other organisms report Cas9-associated toxicity and off-target DNA breaks. Upon the construction of a C. albicans strain collection, we took the opportunity to compare strains which were constructed using CRISPR-Cas9-free and CRISPR-Cas9-dependent transformation strategies, by quantifying and describing transformation-induced loss-of-heterozygosity and hyperploidy events. Our analysis of 57 strains highlights the mutagenic effects of transformation in C. albicans, regardless of the transformation protocol, but also underscores interesting differences in terms of genomic changes between strains obtained using different transformation protocols. Indeed, although strains constructed using the CRISPR-Cas9-free transformation method display numerous concomitant genomic changes randomly distributed throughout their genomes, the use of CRISPR-Cas9 leads to a reduced overall number of genome changes, particularly hyperploidies. Overall, in addition to facilitating strain construction by reducing the number of transformation steps, the CRISPR-Cas9-dependent transformation strategy in C. albicans appears to limit transformation-associated genome changes.

**IMPORTANCE** Genome editing is essential to nearly all research studies aimed at gaining insight into the molecular mechanisms underlying various biological processes, including those in the opportunistic pathogen Candida albicans. The adaptation of the CRISPR-Cas9 system greatly facilitates genome engineering in many organisms. However, our understanding of the effects of CRISPR-Cas9 technology on the biology of C. albicans is limited. In this study, we sought to compare the extents of transformation-induced genomic changes within strains engineered using CRISPR-Cas9-free and CRISPR-Cas9-dependent transformation methods. CRISPR-Cas9-dependent transformation allows one to simultaneously target both homologs and, importantly, appears less mutagenic in C. albicans, since strains engineered using CRISPR-Cas9 display an overall decrease in concomitant genomic changes.

## Abstract (precis)

Genome editing is essential to nearly all research studies aimed at gaining insight into the molecular mechanisms underlying various biological processes, including those in the opportunistic pathogen Candida albicans. The adaptation of the CRISPR-Cas9 system greatly facilitates genome engineering in many organisms. However, our understanding of the effects of CRISPR-Cas9 technology on the biology of C. albicans is limited. In this study, we sought to compare the extents of transformation-induced genomic changes within strains engineered using CRISPR-Cas9-free and CRISPR-Cas9-dependent transformation methods. CRISPR-Cas9-dependent transformation allows one to simultaneously target both homologs and, importantly, appears less mutagenic in C. albicans, since strains engineered using CRISPR-Cas9 display an overall decrease in concomitant genomic changes.

## INTRODUCTION

Candida albicans, often found as a commensal yeast of the human gastrointestinal tract, is also a notorious clinical fungal pathogen. It ranks as the fourth leading cause of nosocomial infections, principally due to the increasing number of immunocompromised individuals (1). Nowadays, C. albicans is arguably the pathogenic yeast that is used to the largest extent in order to investigate fungal pathogenesis at the molecular level. As a consequence, many molecular resources have been generated to facilitate the study of C. albicans biology, including but not restricted to gene knockout and gene overexpression strain collections (2–4), all requiring C. albicans genome editing. Generation of gene knockouts or gene fusions with reporter genes has relied principally on homologous recombination (HR) at target loci using PCR-generated cassettes with 70 to 100 nucleotides of homology to the targeted genomic locus and a lithium acetate-heat shock protocol (5) or an electroporation protocol (6). These strategies allow single-allele disruption, which can quickly become cumbersome in a diploid organism such as C. albicans, since multiple transformation rounds are required for the construction of null mutants. Another inconvenience that has emerged over time is the occurrence of nonspecific genome rearrangements in the course of transformation; several studies have reported transformation-associated genome changes, which sometimes impact the phenotypic traits of engineered strains (7–11). The occurrence of loss-of-heterozygosity (LOH) events and aneuploidies has been described previously and can occur on chromosomes other than those targeted by genetic manipulations (12–15). Interestingly, while a short exposure of C. albicans to heat during the transformation heat shock process tends to increase aneuploidy events, longer exposure to a milder heat favors the appearance of LOH events (14). Previous work has also shown that aneuploid strains are particularly susceptible to undergoing genomic changes upon transformation (14). As a safeguard, researchers have been advised to test the ploidy of their mutants at various genome locations using tools such as the quantitative PCR (qPCR) ploidy screen (13).

The discovery and adaptation of the CRISPR (clustered regularly interspaced short palindromic repeat)-Cas9 system has revolutionized genome editing in a multitude of organisms. Initially described in Streptococcus pyogenes, the CRISPR and its endonuclease Cas9 act as a bacterial defense system against non-self DNA. A single guide RNA (sgRNA) directs the cleaving activity of Cas9 to a specific genomic site. This sgRNA is composed of a recognition motif of 20 bp directly followed by a protospacer-adjacent motif sequence (PAM), composed of the nucleotides NGG (16). Bacterial protection is achieved by recognition and induction of DNA double-strand breaks (DSB) in the non-self DNA (17). The CRISPR-Cas9 technology is the latest of several customizable DNA-binding nucleases that have been engineered to facilitate the introduction of DNA fragments at a specific genomic location thanks to HR upon repair of the induced DNA DSB (18). Its ability to target DSB during genetic manipulations stimulates HR at a specific locus, thus enhancing transformation efficiency over that with classical transformation approaches.

The CRISPR-Cas9 technology has been successfully implemented in mammalian cells, plants, bacteria, and fungi, including C. albicans, and continues to be adapted to an increasing number of organisms. In diploid organisms, such as C. albicans, this technique has facilitated the construction of null mutants, where both alleles may be modified at once (19). CRISPR-Cas9 has also been successfully used to generate multiple simultaneous knockouts using a unique sgRNA to target multiple genes in C. albicans (S. Bachellier-Bassi, personal communication). However, due to its novelty, the repercussions of the active CRISPR-Cas9 system on the biology of C. albicans are not yet fully understood. In Saccharomyces cerevisiae, the integration and constitutive expression of Cas9 have been associated with cell toxicity and lowered fitness of strains, as illustrated by a lower growth rate. Additionally, the toxicity of constitutive Cas9 expression has been associated with a lower transformant yield (20). To limit these effects, a transient system has been developed in C. albicans circumventing constitutive Cas9 and sgRNA expression (21). Another issue is raised by reports of off-target DSB by Cas9 in mammalian cells (22–24). Overall, our understanding of the effects of CRISPR-Cas9 technology in C. albicans is limited, especially in terms of its impact on the integrity of the genome.

We had initially set out to build a collection of isogenic strains possessing an LOH reporter system allowing detection of LOH events throughout the genome of C. albicans. This system involves the insertion of two fluorescent marker genes in a neutral genomic region on both homologs of a given chromosome, allowing spontaneous LOH events to be detected by monitoring the loss of one of the fluorescent markers using flow cytometry. While we were validating our collection of transformed strains, whole-genome sequencing revealed the presence of genome changes. Here, we present a descriptive study of the genome changes in our collection of transformed C. albicans strains, comparing strains constructed using CRISPR-Cas9-free and CRISPR-Cas9-dependent methods. With a total of 57 sequenced strains, our results illustrate and highlight the important mutagenic properties of each transformation protocol both in terms of quantification and in terms of the nature of those genome changes.

Overall, the democratization of whole-genome sequencing allowed us to address the extent of “unwanted” genome changes during strain construction in C. albicans. We show that the CRISPR-Cas9-free transformation method is a high inducer of genome changes, often with multiple genome change events within a single constructed strain, in contrast to the CRISPR-Cas9-dependent transformation method, which results in less-frequent concomitant genome changes.

## RESULTS

### Strain construction and phenotyping.

**FIG 1:**
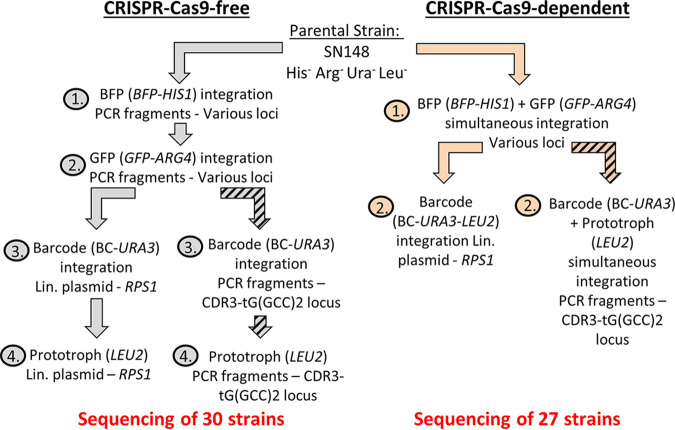
CRISPR-Cas9-free and -dependent transformation strategies used for strain construction. Derived from the C. albicans reference strain SC5314, the parental SN148 strain was sequentially transformed using CRISPR-Cas9-free (gray) or CRISPR-Cas9-dependent (orange) strategy in order to integrate (i) the BFP/GFP LOH reporter system, (ii) a unique barcode sequence, and (iii) the leucine marker rendering the strains prototrophic. Strains constructed using the CRISPR-Cas9-free strategy underwent four transformation steps, as opposed to two using the CRISPR-Cas9-dependent strategy. The barcode sequence (BC) and *LEU2* auxotrophic marker are introduced at the *RPS1* locus on Chr1. The BC and *LEU2* marker are introduced at another locus for the strains carrying the LOH reporter system on Chr1 [in the intergenic sequence between the *CDR3* and tG(GCC)2 loci], as indicated by the striped arrows.

We previously established an LOH reporter system for C. albicans whereby the *GFP* gene (encoding green fluorescent protein) is integrated at a given locus on one chromosome and the *BFP* gene (encoding blue fluorescent protein) is integrated on the homologous chromosome at the same locus (25). LOH at this locus leads to the loss of either the *BFP* or the *GFP* gene, and the frequency at which LOH events arise can be quantified using fluorescence-activated cell sorter (FACS) analysis. In an attempt to explore variations in LOH frequency on the eight C. albicans chromosomes, we undertook to construct a collection of isogenic strains, each possessing the BFP/GFP LOH reporter system at a distinct genomic locus (see Table S1 in the supplemental material). In addition to the LOH reporter system, a unique barcode sequence was also integrated into each strain in order to permit pool experiments. All strains derive from SN148, a C. albicans laboratory reference strain displaying arginine, histidine, uridine, and leucine auxotrophies. This strain was sequentially transformed in order to integrate (i) the BFP/GFP LOH reporter system, (ii) a unique barcode sequence, and (iii) the leucine marker rendering the strains prototrophic. CRISPR-Cas9-free and CRISPR-Cas9-dependent transformation methods were used to generate a total of 57 strains (30 and 27 strains, respectively) (Fig. 1). Both methods are based on homology-directed recombination with exogenous DNA, prepared using PCR and directed to the target genomic locus by the two flanking homology regions (100 bp). Additionally, both the CRISPR-Cas9-free and CRISPR-Cas9-dependent transformation protocols use lithium acetate/polyethylene glycol (PEG) and heat shock treatments to transform the yeast cells. Transformation by electroporation was not considered, since it has been shown to be associated with frequent ectopic integrations of free DNA (6, 26).

10.1128/mSphere.00620-20.2 TABLE S1 C. albicans strains used in this study. Download Table S1, XLSX file, 0.01 MB. Copyright © 2020 Marton et al. 2020 Marton et al. This content is distributed under the terms of the Creative Commons Attribution 4.0 International license.

**FIG 2:**
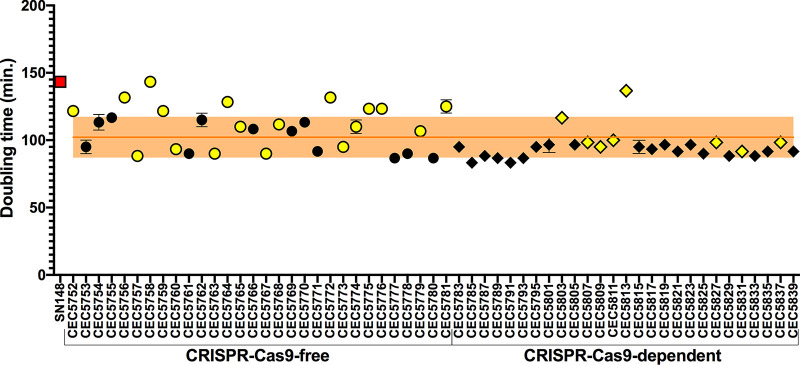
Doubling times in YPD medium at 30°C. Each data point is representative of the average doubling time (in minutes) (*n* = 8) of the parental (SN148) strain (red square) or a constructed strain (circles, CRISPR-Cas9-free method; diamonds, CRISPR-Cas9-dependent method), with error bars indicating standard deviations. The average doubling time of all constructed strains (57 strains) is represented by the orange horizontal line (with orange shading indicating standard deviations). Strains displaying at least one genomic change are shown in yellow, while black indicates strains that are free of transformation-acquired genomic changes.

All engineered strains underwent basic phenotyping to assess if the transformation process drastically impacted their behavior. The functionality of auxotrophy markers associated with integration cassettes was tested by spot assay, where all strains demonstrated a capacity to grow on media with the dropout amino acid histidine, arginine, uridine, or leucine. Moreover, the functionality of the fluorescent proteins BFP and GFP was validated by fluorescence microscopy and flow cytometry. No strain exhibited any difference in colony morphology from the parental strain SN148 on yeast extract-peptone-dextrose (YPD) or synthetic defined (SD) medium. Additionally, the doubling times of all constructed strains were measured in YPD medium at 30°C (Fig. 2). We observed that the parental SN148 strain possesses a longer doubling time, which is most likely due to uridine auxotrophy (27). Among the strains displaying genomic changes (Fig. 2, yellow symbols), some have doubling times longer than the average doubling time obtained for all 57 strains analyzed in this study.

### Genome sequencing.

**FIG 3:**
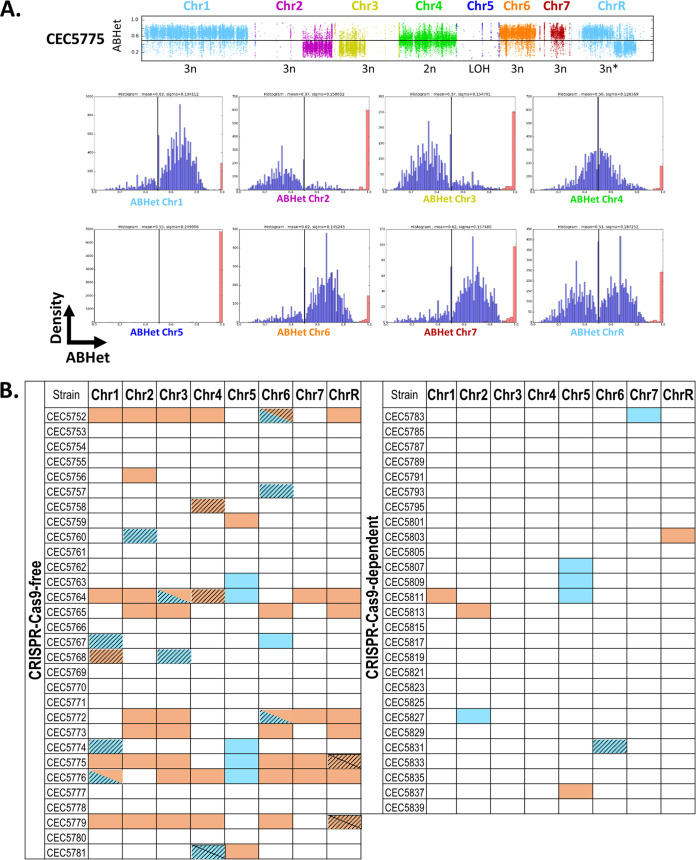
Identification of transformation-induced genome changes within the 57 sequenced C. albicans strains. (A) Determining the average ABHet values per chromosome (Chr) for strain CEC5775. (Upper panel) Plots of allele balances for the eight chromosomes of strain CEC5775. (Lower panels) Histograms illustrate the distribution of ABHet values across a given chromosome, where the black vertical bar represents a 0.5 ABHet value (heterozygous diploid). ABHet and ABHom values are shown in blue and red, respectively. Data interpretation for each chromosome is as follows: Chr1, -6, and -7, trisomy (1×HapA, 2×HapB); Chr2 and -3, trisomy (2×HapA, 1×HapB); Chr4, disomy (1×HapA, 1×HapB); Chr5, LOH; ChrR, recombination event localized in proximity of the centromere plus trisomy (left arm, 1×HapA, 2×HapB; right arm, 2×HapA, 1×HapB). Additional LOH events have been described previously in parental strain SN148 (LOH on Chr2) (25) and in SC5314 (LOH on Chr3 and Chr7) (12). (B) Summary of the genomic changes identified across the eight chromosomes for all 57 sequenced C. albicans strains, using the strategy presented in panel A. The plots showing the allele balance at heterozygous positions and the mean ABHet values per chromosome for each strain can be found in Fig. S1 and Table S5, respectively. LOH events are indicated in blue, aneuploidies in orange. Genomic changes impacting whole chromosomes are identified by solid colors, while those partially impacting chromosomes are identified by a striped pattern.

To ensure that the strains did not acquire major genome changes during transformation, we took the opportunity to perform whole-genome sequencing. In view of time and resource constraints, we took advantage of a pipeline available for rapid genome sequencing of our 57 strains that was calibrated to give 50× coverage for a bacterial genome (4.6 Mb). Based on the C. albicans genome size, 15× coverage was expected, which would still be sufficient to allow detection of LOH and aneuploidies. An average sequencing depth of 25.5 ± 17.2 (ranging from 7.44× to 73.12×) was obtained upon sequencing of the 57 C. albicans strains (Table S4). Postsequencing cleanup and single nucleotide polymorphism (SNP) calling were conducted, and allele ratios at heterozygous sites (ABHet) were evaluated in order to allow identification of genome changes, in particular aneuploidy and LOH. Despite the low sequencing depth, ABHet plots were successfully generated by plotting the allele balance at heterozygous positions across the genome (Fig. S1) and were used to visually identify large-scale LOH events as represented by an absence (or weak presence) of ABHet values. Although less striking in ABHet plots (Fig. S1) due to limited sequencing depth, hyperploidy events (aneuploidy with a chromosome number that is more than the diploid number) were identified by plotting the distribution of ABHet values and calculating the mean ABHet value per chromosome, since hyperploid chromosomes would shift away from the 0.5 ABHet value, which represents a 0.5 allelic ratio (a 50/50 heterozygous nucleotide ratio at the given position). Thus, this method allowed us to differentiate ABHet values corresponding to trisomy (0.33 or 0.66) or tetrasomy (0.25 or 0.75) (Fig. 3A; Table S5). The genomic changes identified throughout the 57 strains are graphically summarized in Fig. 3B.

10.1128/mSphere.00620-20.1 FIG S1 Plots of allele balance at heterozygous sites (ABHet) for the collection of 57 C. albicans strains. Shown are ABHet plots across the eight chromosomes for each of the 57 sequenced C. albicans strains. ABHet values are color-coded as follows: salmon, >0.6; white, <0.6 and >0.4; blue, <0.4. Download FIG S1, JPG file, 2.3 MB. Copyright © 2020 Marton et al. 2020 Marton et al. This content is distributed under the terms of the Creative Commons Attribution 4.0 International license.

10.1128/mSphere.00620-20.5 TABLE S4 Sequencing depth of C. albicans strains. Download Table S4, XLSX file, 0.01 MB. Copyright © 2020 Marton et al. 2020 Marton et al. This content is distributed under the terms of the Creative Commons Attribution 4.0 International license.

10.1128/mSphere.00620-20.6 TABLE S5 Analysis of average ABHet values per chromosome. Download Table S5, XLSX file, 0.01 MB. Copyright © 2020 Marton et al. 2020 Marton et al. This content is distributed under the terms of the Creative Commons Attribution 4.0 International license.

As illustrated by elevated standard deviations (Table S4), an uneven sequencing depth was obtained across our genomes, probably resulting from library preparations being optimized for bacterial genomes. This prevented us from using sequencing depth data to identify or characterize genome changes, and therefore, we could not distinguish monosomies from diploid whole-chromosome LOH events. Since we relied solely on ABHet analysis to study transformation-induced genome changes, we do acknowledge that we may be underestimating the occurrence of aneuploidy events, notably events resulting in a balance of both haplotypes, such as balanced tetraploidy (2×HapA 2×HapB events), which would also be represented as a 0.5 ratio in the ABHet graphs. Nevertheless, this methodology allowed us to efficiently identify numerous genome changes generated during the transformation process.

### Both CRISPR-Cas9-free and CRISPR-Cas9-dependent transformations trigger unwanted genomic changes, but to different extents.

**FIG 4:**
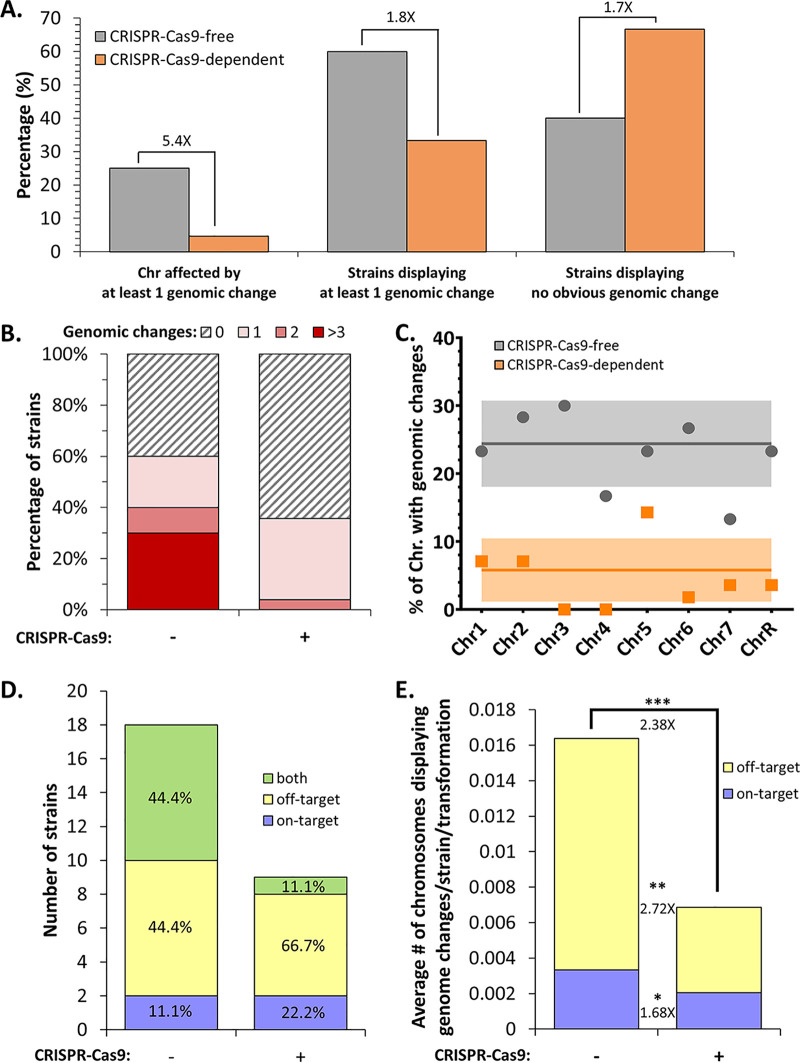
Quantification of genome changes identified within C. albicans strains engineered using one of two transformation methods. (A) Percentages of chromosomes and strains impacted by transformation-associated genomic changes, as well as percentages of genomic-change-free strains. (B) Studying concurrent genomic changes within strains. (C) Percentage of each chromosome affected by at least one genomic change. (D) Representations of the numbers of strains displaying genomic change(s) on a targeted chromosome, a nontargeted chromosome, or both types. The fraction of each category of strains within genomic-change-displaying strains is indicated by a percentage. (E) Frequency of transformation-associated genomic changes tabulated as the average number of chromosomes displaying genome changes per strain per transformation. Differences in genome change frequencies between strains constructed using the two transformation strategies are represented as fold changes (*, *P* < 0.05; **, *P* < 0.01; ***, *P* < 0.001 by *t* test).

**FIG 5:**
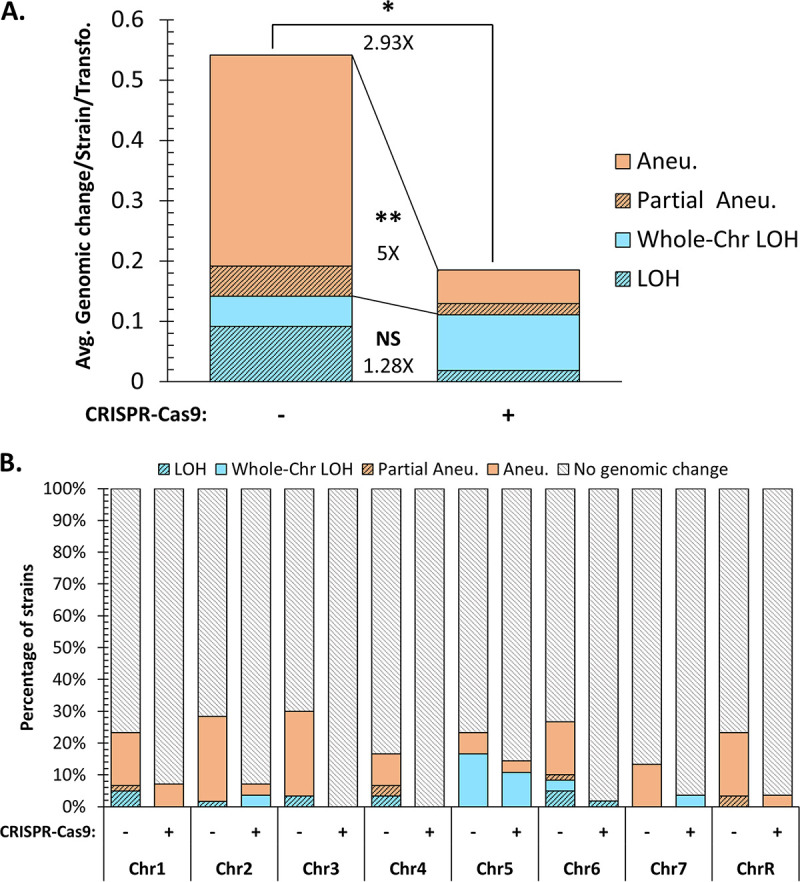
Nature of genomic changes identified within sequenced C. albicans strains. (A) Frequency of transformation-associated genomic changes tabulated as the average number of genome changes per strain per transformation. Differences in genomic change frequencies between the two transformation methods are represented as fold changes (*P*, ≤0.05 by *t* test). (B) Percentage of strains displaying each type of genomic change per chromosome and transformation strategy.

Among the 30 strains constructed using the CRISPR-Cas9-free method, a total of 65 genomic changes were identified (Fig. 3B). Only 40% of the strains (12/30) displayed no obvious genomic changes, while the other 60% (18/30) had at least one genomic change, of which half (9/18 strains) possessed three or more genomic changes (Fig. 4A and B). Of interest, 10/18 strains displayed at least one rearrangement on the targeted chromosome, sometimes accompanied by additional changes on other chromosomes. The remaining 8/18 strains displayed changes only on a chromosome(s) not targeted by the transformation process (Fig. 4D). At the chromosome scale, one-quarter of the sequenced chromosomes (60/240) displayed at least one genomic change, including five chromosomes with two identifiable genomic changes (Fig. 4A; Table S6). Although our analysis did not reveal any obvious enrichment of genomic changes on a given chromosome, we did notice slightly higher and lower numbers of changes on chromosome 4 (Chr4) and Chr7, respectively (Fig. 4C). Overall, strains constructed using the CRISPR-Cas9-free method displayed an average of 0.54 genomic change per strain per transformation (Fig. 5A).

10.1128/mSphere.00620-20.7 TABLE S6 Identification and characterization of genomic changes in the collection of 57 sequenced C. albicans strains. Download Table S6, XLSX file, 0.05 MB. Copyright © 2020 Marton et al. 2020 Marton et al. This content is distributed under the terms of the Creative Commons Attribution 4.0 International license.

Because of the extensive mutagenic effect of CRISPR-Cas9-free transformation, the CRISPR-Cas9-dependent method, via transient Cas9 expression, was favored to generate the second part of our collection, since it reduces the number of sequential transformations (Fig. 1) by targeting DNA DSB on both homologs of the loci of interest. A total of 10 genomic changes, distributed among 9 strains (33.3%), were identified within the 27 strains engineered by this method (Fig. 4A and B). While one-third of the strains (3/9) displayed at least one change on the targeted chromosome, at times accompanied by additional changes on other chromosomes, the others displayed changes only on nontargeted chromosomes (Fig. 4D). At the chromosome scale, only 4.6% of the sequenced chromosomes demonstrated genomic changes (10/216), with no chromosomes displaying multiple changes (Fig. 4A; Table S6). These genomic changes were distributed among most chromosomes, apart from Chr3 and Chr4, which, in our hands, were not affected by changes even when targeted. In contrast, Chr5 seemed to exhibit the highest number of genomic changes (Fig. 4C). We estimated that strains constructed using the transient CRISPR-Cas9 method displayed an average of 0.19 genomic change per strain per transformation (Fig. 5A).

### Aneuploidies are overrepresented in strains engineered using the CRISPR-Cas9-free method.

Genome sequence analysis allowed us to investigate in more detail the nature of the genomic changes observed in the strains engineered using either the CRISPR-Cas9-free or the CRISPR-Cas9-dependent method. The 75 large-scale genomic changes identified above were categorized into two major types: LOH and hyperploidy. Additionally, we report on the sizes of the genome changes, spanning a partial or entire chromosome. Hyperploidies were defined as regions possessing heterozygous SNPs with an allele balance value greater or less than 0.5. For comparison purposes, we took into account the fact that the strain did not go through the same number of transformations in both methods, and we present the data in terms of number of changes per strain per transformation (Fig. 5A).

With the CRISPR-Cas9-free transformation strategy, we observed on average 2.86 times more aneuploidy events (0.40 event) than LOH events (0.14 event) per strain per transformation (*P*, 0.0279 by *t* test). Additionally, both types of genome changes were identified, in decreasing order of abundance, as follows; full chromosome hyperploidies (0.35 event/strain and transformation), partial chromosomal LOH (0.09 event), partial chromosomal hyperploidies (0.05 event), and full-chromosome LOH (0.05 event) (Fig. 5A).

In contrast, strains transformed with the CRISPR-Cas9-dependent transformation strategy displayed comparable average numbers of hyperploidy (0.07 event) and LOH (0.11 event) events per strain per transformation (*P*, 0.493 by *t* test). In decreasing order of abundance, we detected full-chromosome LOH (0.09 event), full-chromosome hyperploidies (0.06 event), partial chromosomal LOH (0.02 event), and partial chromosomal hyperploidy (0.02 event) (Fig. 5A). Our detailed analysis did not reveal any obvious link between the nature of the genomic changes and a specific chromosome, although Chr5 and Chr6 seemed to be implicated in LOH events more frequently than the other chromosomes (Fig. 5B).

## DISCUSSION

The construction of a C. albicans strain collection, aimed at studying genome-wide LOH dynamics, allowed us to retrospectively compare the mutagenic landscapes of strains engineered using two transformation strategies: a CRISPR-Cas9-free and a CRISPR-Cas9-dependent strategy. Whole-genome sequencing of 57 engineered strains of C. albicans permitted the identification and comparison of transformation-acquired genomic changes. Although this study was not designed for this purpose, we observed during the retrospective analysis that C. albicans strains engineered using the CRISPR-Cas9-dependent transformation method displayed significantly fewer concomitant genomic changes, notably in terms of hyperploidy events, than strains constructed using the CRISPR-Cas9-free transformation strategy.

As with transformation protocols of other yeast species, C. albicans competent cells are prepared and suspended in a lithium acetate solution with single-stranded carrier DNA (often single-stranded salmon sperm DNA) and the exogenous DNA, intended to be integrated into the yeast genome. The exogenous DNA usually consists of linearized plasmid DNA with free ends having homology with the yeast genome, or PCR-amplified integration cassettes (5), which are targeted to a specific locus by two homology regions (70 bp to 100 bp) flanking the DNA of interest associated with a selection marker. These homology regions on the free DNA allow strand invasion at the genomic homology site, ultimately resulting in the integration of the repair template through recombination events. Studies have shown that DNA DSB in C. albicans are repaired predominantly via HR and only rarely by nonhomologous end joining (NHEJ) (28–32). This facilitates genetic manipulations of both laboratory and clinical isolates, since efficient genetic modifications are not restricted to NHEJ-deficient strains (as is the case in numerous fungi [33–35]). However, this technique allows the targeting of a single homolog at one locus, i.e., one integration per transformation process, rendering certain strain constructions cumbersome. The development of molecular tools permitting locus-specific DNA DSB has largely facilitated genome editing in numerous eukaryotic organisms by means of zinc finger nucleases (ZFN) or transcription activator-like effector nucleases (TALEN), which recognize a specific DNA sequence and mediate DNA DSB by the FokI nuclease (18). Nevertheless, the most recent and popular targeted genetic modification system is CRISPR-Cas9, because it is highly versatile and its implementation is less laborious than that of the nucleases mentioned above. A sgRNA guides the Cas9 nuclease to its target sequence, where it induces a DNA break. Promoting DNA DSB at the target locus increases HR, resulting in higher transformation efficiency, and allows the integration of exogenous DNA at the target locus on multiple homologs, facilitating genetic engineering in diploid and polyploid organisms.

We observed that both CRISPR-Cas9-free and CRISPR-Cas9-dependent transformations provoke unwanted genomic changes in C. albicans. Our data clearly showed that the transformation process itself is mutagenic, since, by cutting in half the transformation steps required to engineer our strains, we approximately doubled the number of strains free of unwanted transformation-associated genomic changes (Fig. 4A). Despite differences in the number of transformation steps and constructs (use of one versus two barcode/prototrophy constructs [see Materials and Methods]), both transformation strategies produced genomic changes, though to different extents, since C. albicans strains constructed using the CRISPR-Cas9-dependent transformation process displayed obvious reductions in concomitant genomic changes (Fig. 4). Indeed, we observed that 5.4 times fewer chromosomes were affected by undesirable genomic changes among the strains engineered with the CRISPR-Cas9-dependent method. Strains constructed using the CRISPR-Cas9-free strategy displayed multiple coexisting genomic changes; 40% of them carried more than 1 change (up to 8 events), in contrast to 3.7% of strains engineered using the CRISPR-Cas9-dependent method (Fig. 4B). The global mutagenic frequency (average genomic change per strain per transformation) was significantly higher (2.93-fold [*P* = 0.0145]) with the CRISPR-Cas9-free strategy than with the CRISPR-Cas9-dependent method (Fig. 5A). These results suggest that strains engineered by CRISPR-Cas9-free transformations probably derive from cells that endured strong perturbations in genomic stability, thus generating numerous concomitant genomic changes. In contrast, directing DNA DSB with the CRISPR-Cas9-dependent method favors HR-mediated repair and integration of exogenous DNA at the target locus, thus yielding more transformants independently of overall genome perturbation. From a mechanistic perspective, the free linear DNA (repair template) may attempt multiple strand invasions during a homology search, leading to a series of abortive Holliday junctions, perhaps altering overall chromosomal stability, and resulting in large LOH and/or aneuploidies on on- and/or off-target chromosomes. On the other hand, the global mutagenic reduction in strains resulting from CRISPR-Cas9 transformation may potentially be associated with activation of the DNA damage response upon DNA DSB, leading to a global response yielding a cellular environment conducive to repair, including changes in the cell cycle, chromosome mobility, transcription, and nuclear deoxynucleoside triphosphate (dNTP) levels (36).

In other organisms, it has been shown that CRISPR-Cas9 can induce off-target DNA DSB, which may have grave implications (23, 24). To the best of our knowledge, this has not been studied in C. albicans. The genomic changes identified within our strain collection are distributed quasi-equally between the eight chromosomes, and LOH and/or hyperploidies are found predominantly on off-target chromosomes in strains derived from both strategies, even though strains engineered using the CRISPR-Cas9-free strategy are always more impacted by genomic changes (Fig. 4E and 5B). Unfortunately, since genomic changes more frequently involved off-target chromosomes (2.38-fold) (Fig. 4E), we cannot rule out the possibility that they resulted from off-target Cas9 DNA DSB in strains that were generated using the CRISPR-Cas9-dependent strategy. Heat shock is a well-known source of DNA breaks, has been associated with the appearance of genomic changes in C. albicans (37), and could explain the changes observed in our strain set, which experienced a heat shock during the transformation protocol (15 min, 44°C). Further investigations need to be conducted in order to properly address the potential off-target activity of Cas9 in C. albicans.

The genome of C. albicans is highly tolerant of genomic rearrangement events, which often arise upon exposure to various stresses, e.g., heat, fluconazole, or oxidative stress (11, 12, 14, 37). Since transformation protocols involve inflicting stress, it comes as no surprise that our study and these previous studies revealed the presence of LOH and aneuploidy events in various laboratory strains. Indeed, microarray experiments and haplotype mapping have highlighted the mutagenic effect of transformation in multiple laboratory backgrounds (RM1000, CAI-4, and BWP17) between strain stocks and their derivatives (12–14, 38). For instance, transformation of SC5314 to generate the uracil auxotrophic CAI-4 strain (39) has been linked to trisomy of Chr1 (10) or Chr2 (11), while *HIS1* disruption in CAI-4 resulted in deletion of telomere-proximal genes of Chr5 in its derivative BWP17 (11, 40). The same was true among our collection of 57 transformed strains, for which we mainly observed two sorts of genomic changes: (i) LOH and (ii) hyperploidies (defined in our study as a chromosome or a portion of a chromosome present in more than two copies). In our collection, transformation-induced LOH events appeared at comparable rates of 0.14 and 0.11 LOH event/strain/transformation in strains constructed using CRISPR-Cas9-free and CRISPR-Cas9-dependent methods, respectively (1.28-fold change [*P*, 0.5715 by *t* test]). In contrast, strains obtained using the CRISPR-Cas9-dependent strategy displayed a 5-fold decrease (*P*, 0.0086 by *t* test) in hyperploidy events relative to the strains constructed using the CRISPR-Cas9-free method (Fig. 5A). Frequent chromosome trisomies were observed in our strain set (see Tables S5 and S6 in the supplemental material). Chromosome trisomies have often been associated with rapid adaptation to stress conditions, such as that demonstrated by increased fitness in the presence of fluconazole upon Chr5 aneuploidy (41, 42). Additionally, genotypic and phenotypic diversification, namely, Chr5 and Chr6 trisomies, has been described upon exposure of C. albicans to the oral niche, leading to a low-virulence phenotype, suggesting an adaptation resulting in a commensal-like phenotype (43, 44).

Taking our findings together, transformation protocols limiting the number of sequential transformation steps should be favored in order to minimize the acquisition of unwanted transformation-associated genomic changes. Additionally, we want to highlight the importance of generating, testing, and analyzing multiple transformants, since, at best, one-third of transformed strains undergo genomic changes (Fig. 4A and B). Though ideal, routine whole-genome sequencing of transformed strains is not necessarily feasible. Alternatives have been proposed by others, such as a qPCR-based assay to monitor copy number variations throughout the genome of C. albicans (13). However, this technique is limited to the identification of aneuploidies and does not permit the detection of equally important LOH events. The SNP-restriction fragment length polymorphism (RFLP) technique, allowing one to monitor the allelic status for multiple genomic sites (45), can be a complementary means to detect LOH events. Additionally, recomplementation experiments are key in reverse genetics, allowing one to reinforce the conclusions drawn from phenotypic observations.

To conclude, this study has permitted a detailed investigation of the frequency and nature of genomic changes that occur upon the transformation of C. albicans cells, comparing transformation-induced mutagenic landscapes in strains constructed with two transformation methods: a CRISPR-Cas9-free and a CRISPR-Cas9-dependent strategy. By investigating only large genomic rearrangements, we highlight the fact that C. albicans transformation is highly mutagenic and recognize that we may be vastly underestimating this mutagenic effect; for example, we did not investigate the presence of indels and SNPs. Nevertheless, the CRISPR-Cas9-dependent strategy seems to reduce transformation-associated concomitant genomic changes, especially with regard to hyperploidy events.

## MATERIALS AND METHODS

### Strains and culture conditions.

The yeast strains described in the study were constructed starting from C. albicans strain SN148 (His^–^ Arg^–^ Ura^–^ Leu^–^) (46). Yeast cells were cultured on/in rich YPD medium (1% yeast extract, 2% peptone, 2% dextrose). synthetic defined (SD) medium (0.67% yeast nitrogen base without amino acids, 2% dextrose), and synthetic complete (SC) medium (0.67% yeast nitrogen base without amino acids, 2% dextrose, 0.08% dropout mix with all the essential amino acids), which were used for selection. Solid media were obtained by adding 2% agar.

Cloning experiments were conducted using One Shot TOP10 chemically competent Escherichia coli K-12 cells (Thermo Fisher Scientific). E. coli strains were cultured on/in LB (1% Bacto tryptone, 0.5% Bacto yeast extract, 0.5% sodium chloride) or 2YT (1.6% Bacto tryptone, 1% Bacto yeast extract, 0.5% sodium chloride, 0.1% d-glucose) medium, with appropriate antibiotics for selection purposes (50 μg/ml kanamycin, 50 μg/ml ticarcillin). Solid media were obtained by adding 2% agar.

All C. albicans strains and E. coli plasmids are listed in Tables S1 and S2 in the supplemental material, respectively.

10.1128/mSphere.00620-20.3 TABLE S2 Plasmids used in this study. Download Table S2, XLSX file, 0.01 MB. Copyright © 2020 Marton et al. 2020 Marton et al. This content is distributed under the terms of the Creative Commons Attribution 4.0 International license.

### CRISPR-Cas9-free transformation.

Strains constructed using the following CRISPR-Cas9-free protocol underwent four sequential heat shock and lithium acetate/PEG rounds of transformations (47) in order to (i and ii) integrate the BFP/GFP LOH reporter system at a distinct genomic locus, (iii) integrate a unique barcode associated with the *URA3* auxotrophic marker at the *RPS1* locus, and finally (iv) integrate the *LEU2* auxotrophic marker at the *RPS1* locus, rendering the strains prototrophic (Fig. 1). The strategy was to integrate the BFP/GFP LOH reporter system into the most telomere-proximal intergenic region of ≥5 kb on each chromosome arm (Table S1). For this purpose, 120-bp primers were designed, each composed of 20 bp complementary to both the P*_TDH_*_3_-*GFP-ARG4* and P*_TDH_*_3_-*BFP-HIS1* cassettes and 100-bp tails possessing the complementary sequences of the targeted integration locus (Table S3). Each primer pair was used to amplify both the P*_TDH_*_3_-*GFP-ARG4* and P*_TDH_*_3_-*BFP-HIS1* cassettes from plasmids pCRBluntII-P*_TDH3_*-*GFP*-*ARG4* and pCRBluntII-P*_TDH3_*-*BFP*-Cd*HIS1*, respectively. Each cassette was amplified in a total PCR volume of 500 μl, precipitated in 100% ethanol, and resuspended in 100 μl of distilled sterile water. For each transformation, competent cells were transformed with approximately 5 μg of the appropriate DNA cassette. The parental SN148 strain was initially transformed with the P*_TDH_*_3_-*GFP-ARG4* cassette and then subjected to a second round of transformation with the P*_TDH_*_3_-*BFP-HIS1* cassette. These two transformation steps allowed the integration of the BFP/GFP LOH reporter system at a given intergenic locus (Table S1). The resulting strains, except those possessing the BFP/GFP LOH reporter system on Chr1, were then retransformed with StuI-linearized CIp10-P*_TET_*-BC-*URA3* plasmids, each containing a unique barcode (BC) sequence and targeting the C. albicans *RPS1* locus on Chr1. These plasmids are derived from a private laboratory collection of Cip10-P*_TET_*-BC-GTW-*URA3* plasmids (unpublished data), each possessing a unique 25-nucleotide barcode sequence. The Gateway (GTW) cassette was removed by HindIII digestion and self-ligation in order to ensure that its presence did not influence the biology of C. albicans. Last, BFP/GFP barcoded strains were rendered prototrophic by a fourth round of transformation involving the integration of the StuI-linearized CIp10-*LEU2* plasmid at the *RPS1* locus. Conversely, in strains bearing the BFP/GFP LOH reporter system on Chr1, the BC-*URA3* and *LEU2* cassettes were integrated on Chr4 at the CDR3-tG(GCC)2 locus to avoid loss of the latter upon LOH. For these strains, the BC-*URA3* and *LEU2* cassettes were generated using the same PCR amplification protocol described above for the P*_TDH3_*-*GFP-ARG4* and P*_TDH3_*-*BFP-HIS1* cassettes, where long-tailed primers (Table S3) were used for amplification from plasmids CIp10-P*_TET_*-BC-*URA3* and CIp10-*LEU2*, respectively. Throughout the strain construction process, selective pressure was always maintained in order to ensure the selection of transformants carrying all integration cassettes. In addition, at each transformation step, junction PCRs were conducted to ensure the proper integration of cassettes using the primers listed in Table S3.

10.1128/mSphere.00620-20.4 TABLE S3 List of primers. Download Table S3, XLSX file, 0.02 MB. Copyright © 2020 Marton et al. 2020 Marton et al. This content is distributed under the terms of the Creative Commons Attribution 4.0 International license.

### CRISPR-Cas9-dependent transformation.

In contrast to the CRISPR-Cas9-free transformation method, both homologs may be simultaneously targeted for cassette integration with the CRISPR-Cas9-dependent transformation protocol. Thus, by directing a DNA DSB with a locus-specific sgRNA, the BFP/GFP LOH reporter system can be introduced with only one exposure to heat shock and lithium acetate/PEG, rather than two treatments. Hence, only two transformation rounds were required for strain construction, where (i) the BFP/GFP LOH reporter system was integrated at distinct genomic loci and (ii) strains were simultaneously barcoded and rendered prototrophic using both *URA3* and *LEU2* auxotrophic markers (Fig. 1). A total of 11 unique 20-bp sgRNAs were designed using CHOPCHOP (48), targeting the same integration loci as those chosen in the CRISPR-Cas9-free protocol for integration of the BFP/GFP LOH reporter system (Table S3). Because the CIp10-derived BC-*URA3*-*LEU2* plasmids could not be targeted to the *RPS1* locus on Chr1 in the strains bearing the BFP/GFP LOH reporter system on Chr1, an additional sgRNA was designed to target the BC-*URA3* and *LEU2* markers on Chr4 at the CDR3-tG(GCC)2 locus (Table S3) in these strains.

We used a transient CRISPR-Cas9 system (21), which does not necessitate the genomic integration of either Cas9 or sgRNAs. The construction of sgRNAs and the amplification of Cas9 cassettes from the pV1093 plasmid were conducted as described by Min et al. (21), while *BFP-HIS1*-, *GFP-ARG4*-, BC-*URA3*-, and *LEU2*-bearing cassettes were constructed as described above. SN148 cells were cotransformed with 3 μg of the P*_TDH_*_3_-*GFP-ARG4* cassette, 3 μg of the P*_TDH_*_3_-*BFP-HIS1* cassette, 1 μg of the Cas9 cassette, and 1 μg of sgRNA using the lithium acetate/PEG transformation protocol. Transformants were then selected on SC-Arg-His medium, and junction PCRs were performed in order to ensure proper integration of both cassettes at the targeted locus. Strains bearing the BFP/GFP LOH reporter system on Chr1 were then transformed using the transient CRISPR-Cas9 system targeting BC-*URA3* and *LEU2* cassette integration at the CDR3-tG(GCC)2 locus on Chr4. For the remaining strains, both BC-*URA3* and *LEU2* markers were integrated in one transformation step. We did this using the Gateway recombination system, where the *LEU2* gene was transferred from a pDONR-*LEU2* plasmid into Cip10-P*_TET_*-BC-GTW-*URA3* plasmids (unpublished data) by an LR reaction. The unique Cip10-P*_TET_*-BC-*LEU2*-*URA3* plasmids were then linearized by StuI and integrated at the *RPS1* locus. These transformants were selected on SD medium, and junction PCRs were performed.

### Strain phenotyping.

All selected strains underwent basic phenotypic characterization upon validation of cassette integration at targeted loci by junction PCRs. The functionality of the auxotrophic markers was evaluated by drop tests on SC medium with the appropriate dropout amino acid mix, based on the marker tested. Overnight-saturated cultures of selected strains in liquid YPD medium were spotted onto solid YPD, SC-His, SC-Arg, SC-Ura, and SC-Leu media and were placed at 30°C for 24 h to monitor growth. Furthermore, the functionality/intensity of both fluorescent proteins (BFP and GFP) was validated by flow cytometry (MACSQuant analyzer [Miltenyi Biotec]) and fluorescence microscopy (Olympus IX83). The colony morphology of all strains was also assessed on both solid YPD and SD media at 30°C. Finally, doubling times were evaluated in liquid YPD medium at 30°C by measuring the optical density with a Tecan Infinite system over a 24-h period.

### DNA extraction and whole-genome sequencing of strains.

Prototrophic strains were cultured in 5 ml of liquid SD medium overnight at 30°C, and DNA was extracted by following the manufacturer’s protocol using the Qiagen QIAamp DNA minikit. The DNA was eluted in a total volume of 100 μl. The genomes were sequenced at the P2M Platform of Institut Pasteur by using the Illumina sequencing technology. Libraries were prepared with the Nextera XT sequencing kit, and NextSeq500 platforms were used to generate 151-bp paired-end reads.

### Identification of gross chromosomal rearrangements.

Sequences and genomic variations were analyzed as described in references 49 and 50. Each set of paired-end reads was mapped against the C. albicans reference genome, SC5314 haplotype A and haplotype B (version A22-s07-m01-r57), using Minimap2 (51). SAMtools, version 1.9, and Picard tools, version 2.8.1 (http://broadinstitute.github.io/picard), were then used to filter, sort, and convert SAM files. SNPs were called using the Genome Analysis Toolkit (GATK), version 3.6, according to GATK best practices. SNPs were filtered using the following parameters: VariantFiltration, QD < 2.0, LowQD, ReadPosRankSum < –8.0, LowRankSum, FS > 60.0, HightFS, MQRankSum < –12.5, MQRankSum, MQ < 40.0, LowMQ, HaplotypeScore > 13.0. Sequencing depths were also calculated using the Genome Analysis Toolkit (Table S4). The GATK variant filtration walker (VariantAnnotator) was used to add allele balance information to VCF files. The value of allele balance at heterozygous sites (ABHet) is a number that varies between 0 and 1. ABHet is calculated as the number of reference reads from individuals with heterozygous genotypes divided by the total number of reads from such individuals. Thus, a diploid genome will be defined by an ABHet value of 0.5. In contrast, while a triploid strain may contain either three identical alleles (an allelic frequency of 1) or two identical alleles and one different allele (frequencies of 0.66 and 0.33), a tetraploid strain may have allelic frequencies of either 0.5 (2 × 2 identical alleles), 1 (4 identical alleles), or 0.25 and 0.75 (3 identical alleles and 1 different allele). In order to obtain an average ABHet value per chromosome, we evaluated the ABHet and allele balance at homozygous positions (ABHom) with the AlleleBalance annotation GATK module (Table S5). Histograms were built based on the number of SNPs with ABHet values with the matplotlib 2D graphics package (52), with blue and red representing ABHet and ABHom values, respectively.

### Data availability.

Genome sequences of the 57 engineered C. albicans isolates described in this study have been deposited in the NCBI Sequence Read Archive under BioProject ID PRJNA659611. All other relevant data are available from the corresponding author upon request.
