# Supplementary figures and images for: Use of CRISPR-Cas9 To Target Homologous Recombination Limits Transformation-Induced Genomic Changes in Candida albicans
Source: mSphere. 2020 Sep 2;5(5):e00620-20. doi: 10.1128/mSphere.00620-20 (PMC7471004; doi:10.1128/mSphere.00620-20)

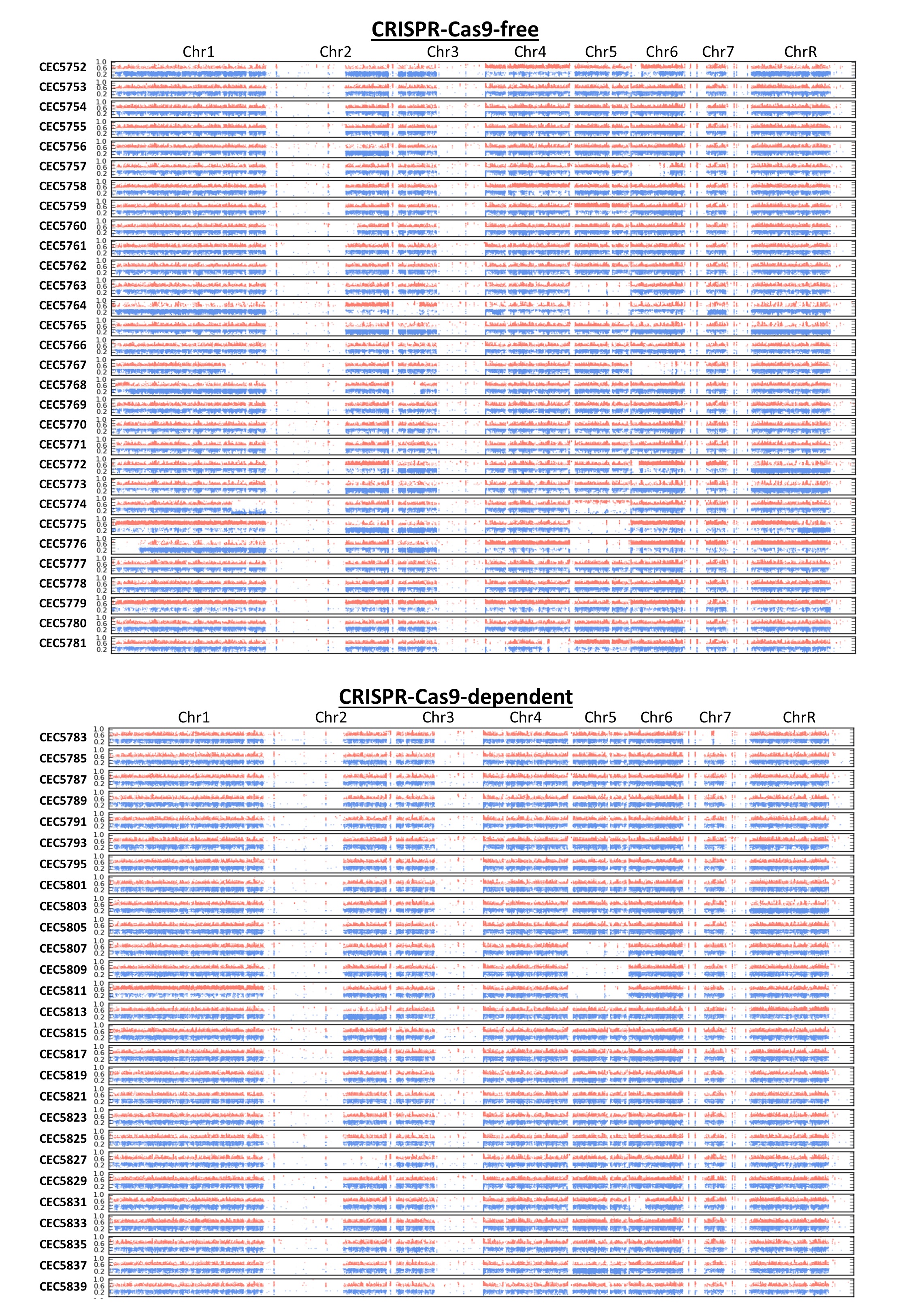

Supplement: FIG S1 [file mSphere.00620-20-sf001.jpg]
